# Application of lecture-and-team-based learning in stomatology: in-class and online

**DOI:** 10.1186/s12909-024-05235-2

**Published:** 2024-03-08

**Authors:** Biyao Wang, Shan Jin, Minghao Huang, Kaige Zhang, Qing Zhou, Xinwen Zhang, Xu Yan

**Affiliations:** 1 The VIP Department, School and Hospital of Stomatology, China Medical University, Liaoning Provincial Key Laboratory of Oral Diseases, Shenyang, China; 2 Department of Oral and Maxillofacial Surgery, School and Hospital of Stomatology, China Medical University, Liaoning Provincial Key Laboratory of Oral Diseases, Shenyang, China; 3 Department of Oral Implantology, School and Hospital of Stomatology, China Medical University, Liaoning Provincial Key Laboratory of Oral Diseases, Shenyang, China; 4 Laboratory Animal Centre, School and Hospital of Stomatology, China Medical University, Shenyang, Liaoning China

**Keywords:** Lecture-based learning, Team-based learning, Lecture-team-based learning, Stomatology, Chinese undergraduate

## Abstract

**Background:**

This study aimed to evaluate stomatological students’ learning efficacy and their attitude towards Lecture-Team-Based Learning (LTBL) on topics regarding the design of removable partial dentures via in-class, online, and both in combination.

**Methods:**

Students from seven distinct grades participated in the course in their fourth academic year (Years 2015, 2016, 2017, 2018, 2019, 2020, and 2021). Students of Years 2015–2019 attended in-class LTBL, students of Year 2020 attended online LTBL, and students of Year 2021 attended the combination mode. The scores of three examinations were compared, namely, individual readiness assessment test, team readiness assurance test, and individual application test. Visual Analog Scales (VAS) were used for students to self-assess their mastery of prosthodontics knowledge before and after the course. Anonymous questionnaires were delivered to evaluate their satisfaction with LTBL via a Likert scale.

**Results:**

In each academic year, the three exam scores were significantly improved as the course progressed and VAS-post scores were significantly higher than VAS-pre scores. The three examination and VAS scores of students in Year 2020 were significantly lower than those in Years 2019 and 2021. Students were highly satisfied with the LTBL course based on the three parameters of knowledge acquisition, teamwork, and classroom atmosphere.

**Conclusion:**

Students were highly satisfied with the LTBL course and their learning performance was improved as the course progressed both in-class and online. Online LTBL could be adopted when students have to study online, while in-class LTBL could perform better when combined with video records of an online LTBL course.

## Background

Since stomatology has entered the digital medical era, to become a qualified dentist, it is necessary to master not only the knowledge of the oral disciplines, but also the knowledge of mathematics, electronics, informatics, materials science, mechanical engineering and other disciplines. At the begining of theoretical learning, it is hard for students to connect the above disciplines to form an overall medical thinking, which makes stomatology is difficult to combine with clinical practice [1]. Lecture-Based Learning (LBL) is a “teacher-centered teaching model”, with advantages of saving teaching resources and imparting knowledge in an accurate, systematic and coherent way [2], which can help students lay a solid theoretical foundation. Our previous research found that students are more willing to accept LBL than other teaching methods [3]. However, LBL also has disadvantages that students receive information passively from instructors, lacking motivation and active thinking, failing to train them to solve practical problems with their theoretical knowledge [4]. Therefore, a major current problem in stomatological education is how teachers can help students to transform their theoretical knowledge into practical applications.

Team-Based Learning (TBL) is a “student-centered teaching model” developed by Professor Larry Michaelsen [5]. TBL has lots of advantages, which mobilizes students’ initiative and enthusiasm, promotes students’ comprehensive qualities and improves students’ ability to use the knowledge they have learned [6]. TBL promotes the use of team resources for autonomous learning by students through group discussion, so as to cultivate students into lifelong learners. TBL has been positively appraised in colleges of dentistry worldwide, such as at Princess Nourah bint Abdulrahman University, Qassim University, and Seoul National University [7–9]. It has been demonstrated that students’ problem-solving ability has been promoted and they enjoyed the interactive atmosphere provided by team discussions, which helped them to recall the knowledge that they obtained. In order to provide timely and frequent feedback to students, TBL curriculum usually includes following test: the individual readiness assurance test (iRAT), team readiness assurance test (tRAT), and individual application test (iAT) [10]. iRAT is used to evaluate students’ mastery of knowledge before TBL class, while tRAT is used to evaluate students’ teamwork learning efficacy. After TBL class, students work together to solve the clinical case problems and then are given iAT to evaluate the whole course efficacy. This immediate feedback ensured that students are provided with an understanding of their level of content knowledge and encouraged students’ accountability and enthusiasm [11]. Nevertheless, TBL also has its disadvantages, including increasing the burden of learning, weakness of the theoretical knowledge, and students failing to keep up with the progress of the class, which prevents the advantages of TBL from being maximized [12]. Meanwhile, students in other medical disciplines rated the effect of TBL as moderate and some of them became anxious because the learning responsibilities were placed on them [13–15]. To resolve these problems, a new teaching mode combining LBL and TBL has been created: Lecture-Team-Based Learning (LTBL).

In-class LTBL was conducted in prosthodontics courses at the School of Stomatology, China Medical University, Liaoning, China, in 2015–2019 until the outbreak of the Coronavirus Disease 2019 (COVID-19) pandemic. COVID-19 forced students to learn online. Despite the advantages of being unrestricted in terms of time and space, online classes lack interaction between teachers and students and among students themselves [16]. Moreover, a high level of self-control is required due to the lack of teachers’ supervision [17]. Thus, a new teaching method was introduced in 2020, involving the adaptation of LTBL to online learning, in order to make students pay more attention and stimulate their enthusiasm for the classes. In 2021, we had the chance to conduct in-class LTBL and we provided the students with the class videos of 2020 as materials to preview before the class and review after the class. This approach can be regarded as a combination of online and in-class LTBL.

This study aims to assess students’ performance in and satisfaction with LTBL in-class, online, and both in combination. The null hypothesis to be tested in this study is that LTBL has no effect on the students’ performance and satisfaction regarding the following parameters: knowledge acquisition, teamwork, and classroom atmosphere.

## Method

Dentistry undergraduates from the School of Stomatology, China Medical University, who began their first academic year from 2011 to 2017 participated in this study. Inclusion criteria for participants: Students included in the study were enrolled in the prosthodontics course as fourth-year medical students and passed all the exams of the previous three years’ courses. Students of seven distinct grades participated in the course in their fourth academic year (Years 2015, 2016, 2017, 2018, 2019, 2020, and 2021). Exclusion criteria for participants: Students who dropped out of the discipline or withdrew for any reason during the time of this study were excluded from the study. All subjects participated voluntarily in the study. c2 tests and Bonferroni adjustments were used for comparing the male to female ratio. Age and exams score of past three academic years were analyzed and presented as mean ± SD and one-way analysis of variance (ANOVA) was used for testing the significance. A p-value of < 0.05 was considered significant. Data were analyzed using SPSS version 26.0.

**Fig. 1:**
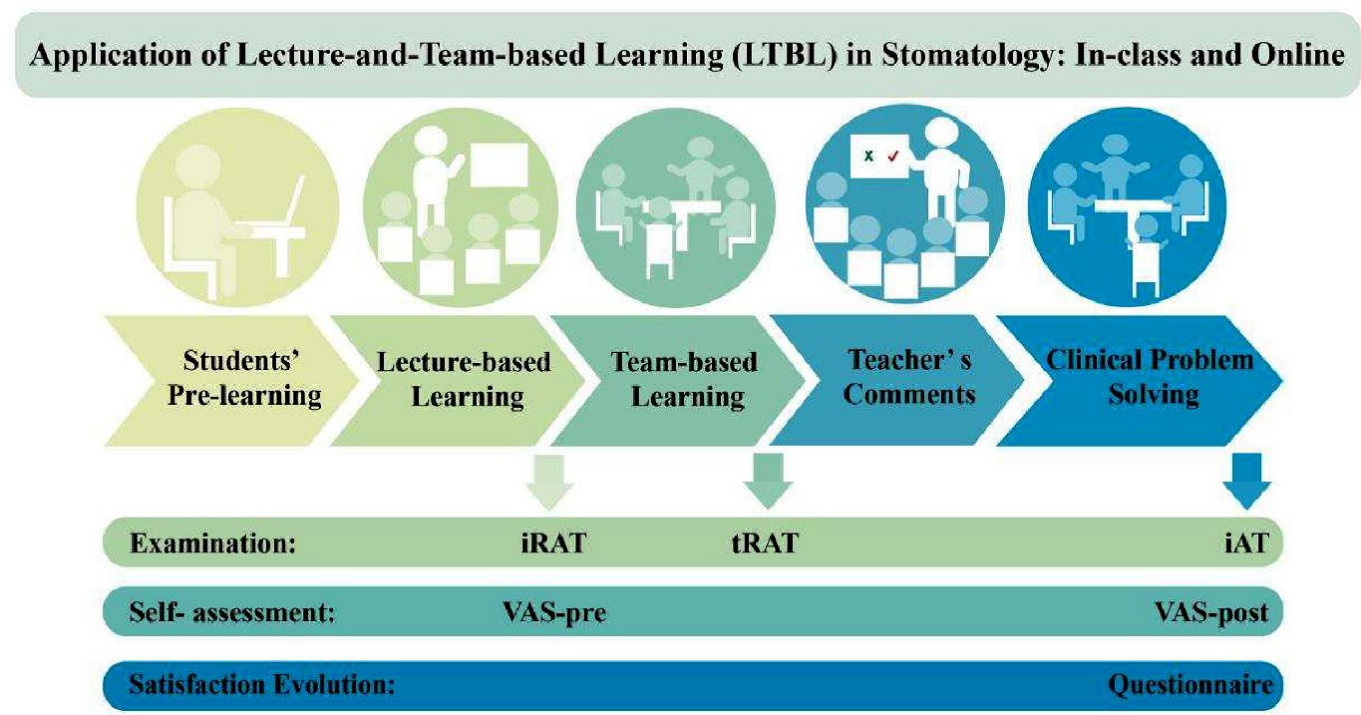
The process of lecture-and-team-based learning

This study was conducted in accordance with the Declaration of Helsinki, and the protocol was approved by the Medical Ethics Committee of the Hospital of Stomatology of China Medical University (2022; No. 13). Figure 1 shows the process of Lecture-and-Team-Based Learning.

### Lecture-and-team-based learning: in-class (years 2015–2019)

#### Phase 1: Lecture-based learning

One week before the scheduled TBL, we explained the procedure and objectives of TBL to the students in order to make them fully understand it. The students were asked to spend 1 week previewing removable partial dentures (RPD) design by reading a series of texts and researching online outside of class. Then, the teacher presented the critical points of RPD design via a PowerPoint presentation and showed designs for specific cases to the students. The curriculum consisted of RPD design studies that covered all categories within the Kennedy classification, which is a worldwide classification of dentition defects. The Kennedy classification consists of four classes. classes I to III are subdivided into modifications, with each modification denoting an additional saddle area. Students were asked to review the knowledge that they had obtained after the class and TBL was conducted 1 week later.

#### Phase 2: Team-based learning

On the day of TBL, each student first completed an individual readiness assessment test (iRAT) in 10 min at the end of the class. The 100-point iRAT consisted of six case-based multiple-choice questions (MCQs) (each question worth 10 points) and one RPD design based on different Kennedy dentition defects (40 points). Overall, 64 types of Kennedy dentition defects were presented on the test papers, including the four classes and their subcategorized modifications, which were numbered as 1-A to D for maxillary Kennedy class I and 1-a to d for mandibular Kennedy class I (and similarly for the other three Kennedy classes). The numbers were given on the test paper and the students were randomly divided into eight teams from A to H (students with the same letter were in one team), which guaranteed that each group could practice all types of Kennedy dentition defects. Meanwhile, students were required to carry out a self-assessment of their prosthodontics knowledge via VAS, recorded as VAS-pre.

Next, the assigned teams completed the team readiness assurance test (tRAT) by discussing the iRAT questions, with answers decided by consensus. They drew their design on a poster, and the teacher delivered a micro-lecture, commenting on each team’s design and summarizing the key points of the curriculum.

#### Phase 3: Clinical problem solving

At the end of the TBL class, eight fictional clinical case problems were randomly distributed to each team. The students were required to design a detailed treatment plan for the patients with dentition defects and each team was then to give a presentation via PowerPoint in the next class a week later. After the students’ presentation, the teacher provided feedback and showed the treatment plan for each case.

To assess individual students’ ability to apply course concepts gained from each module, the students completed an individual application test (iAT) that was specifically related to the case study modules. This took place 2 weeks after the clinical problem-solving class was performed. The 100-point iAT consisted of six case-based multiple-choice questions (each question worth 10 points) and one RPD design (40 points). Students were again required to carry out self-assessment of their prosthodontics knowledge via VAS, recorded as VAS-post.

### Lecture-and-team-based learning: online (Year 2020)

Facing the challenges posed by the COVID-19 pandemic, we conducted LTBL online via a web platform called Rain classroom in 2020 for the students whose first academic year was 2016. The online progress was similar to that of the in-class LTBL. For phase 1 (Lecture-Based Learning), students joined the online classroom where they could not only attend the lecture but also receive and submit their assignments (iRAT, tRAT, and iAT). For phase 2 (Team-Based learning) and phase 3 (Clinical problem solving), students could communicate with their teacher and classmates and gave their presentations via a web conference platform called Tecent Meeting. The teacher could download these materials and arrange them into a PowerPoint presentation to provide feedback and a conclusion via this web platform. All of the classes were recorded by the web platform and could be downloaded by the students for review.

### Lecture-and-team-based learning: combination of online and in-class (Year 2021)

In 2021, the students whose first academic year was 2017 were given in-class LTBL. The progress was similar to that of the previous in-class routine. The difference was that students could also download the video records of Year 2020 for preview before the classes and review them after the classes, although this was not enforceable.

### Assessment and evaluation

#### Examination

We regarded the examination performance of each student as the primary outcome. The iRATs and iATs were graded individually and tRATs were graded for each group. Three reviewers graded the RPD design drawing, and the final grades of the three reviewers were averaged. Exam scores were analyzed and presented as M (P25, P75). Shapiro-Wilk test was used to evaluate normality. The assumption of normality was not confirmed, so non-parametric tests were performed. Wilcoxon signed rank tests were used to examine differences between iRAT and tRAT, iRAT and iAT, and tRAT and iAT scores in each year. Kruskal-Wallis tests were used to examine differences in iRAT, tRAT, and iAT scores among Years 2019–2021, while Kruskal-Wallis one-way ANOVA was used to examine differences between groups. A p-value of < 0.05 was considered significant. Data were analyzed using SPSS version 26.0.

#### Self-assessment

We considered the self-assessment results of each student as the secondary outcome. To promote the validity of the self-assessment [18], we chose to anonymize the assessment process. Specifically, each student was given a number, corresponding to the name of the self-report saved on the computer. The steps above were completed by two teachers outside our research group, who were blinded to the process. Students were required to carry out self-assessments of their prosthodontics knowledge via VAS, which were recorded as VAS-pre and VAS-post. The students were given a horizontal line of 10 cm in length, the ends of which were marked with “0” and “10.” Then, they were asked to mark a position on the line to indicate “How well have you mastered prosthodontics knowledge at this moment?” The distance between the mark and the end representing 0 points reflected the extent of the students’ mastery of prosthodontics knowledge, in their own opinion. The results were considered to represent the self-assessed confidence of the subjects in their own knowledge. The results were analyzed and presented as mean (SD). The collected data were analyzed for a normal distribution using the Shapiro-Wilk test and for homogeneity of variance using Levene’s test. The data were confirmed to be normally distributed. Paired t tests were used to examine differences between VAS-pre and VAS-post in each year. One-way ANOVA was used to examine differences in VAS-pre and VAS-post among Years 2019–2021. When homogeneity of variance was not present, LSD test was used for multiple comparisons. A p-value of < 0.05 was considered significant. Data were analyzed using SPSS version 26.0.

#### Questionnaire

Anonymous questionnaires were delivered to the students to evaluate their satisfaction with LTBL. The questionnaire consisted of 14 questions covering three parameters: knowledge acquisition, teamwork, and classroom atmosphere. Five additional questions were set for the students who participated in online LTBL (2020), and another two questions were set for students who participated in the combination of online and in-class LTBL (2021). Answers were presented on a five-point Likert scale ranging from 1 to 5 (5 = strongly agree, 4 = agree, 3 = neutral, 2 = disagree, and 1 = strongly disagree). Cronbach’ s alpha coefficient tests was used to assess the reliability of the construct. Exploratory factor analysis was used to assess the validity of the construct. To analyze the results of the questionnaire survey, we calculated the mean score for each question. Measurement data were presented as mean (standard deviation). A p-value of < 0.05 was considered significant. Data were analyzed using SPSS version 26.0.

## Result

The numbers of attendees of LTBL sessions ranged from 60 to 64, with a total of 433 resident encounters. Basic demographic information about the participants are displayed in Table 1. There was no significant difference in gender distribution, age and mean score of past three academic years among these students from seven distinct grades, respectively (*P* > 0.05).

**Table 1:** Basic statistics of students from seven distinct grades

	Total Attended	Male/female	Age (years)	Exam score of past three academic years
2015	60	22/38	21.22 ± 0.64	76.17 ± 3.13
2016	63	24/39	21.35 ± 0.83	75.92 ± 3.57
2017	62	22/40	21.26 ± 0.70	76.10 ± 3.33
2018	64	25/39	21.29 ± 0.77	76.38 ± 4.07
2019	61	23/38	21.27 ± 0.61	75.78 ± 2.53
2020	60	21/39	21.35 ± 0.88	76.45 ± 2.46
2021	63	22/41	21.32 ± 0.80	76.59 ± 2.69
		*P* = 0.997	*P* = 0.953	*P* = 0.793

Age and exam scores were reported as mean ± SD

### Score

**Fig. 2:**
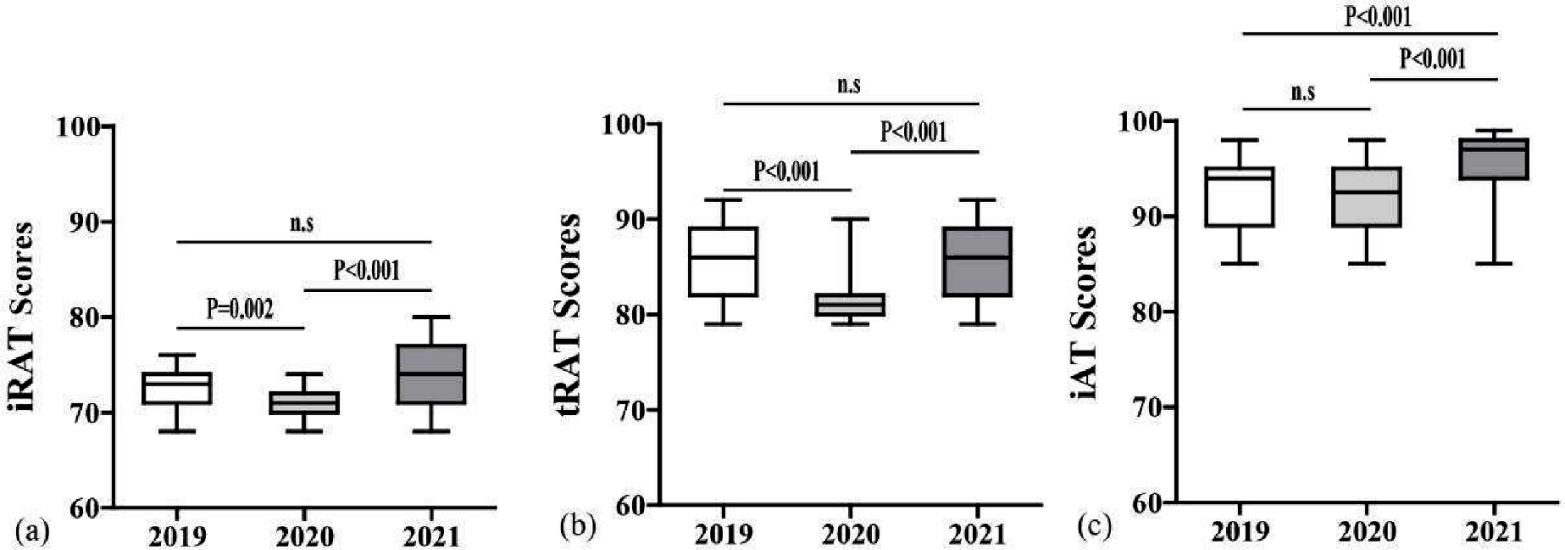
Box-and-whisker diagram of the iRAT (**a**), tRAT (**b**), and iAT (**c**) exam scores across Years 2019–2021, presenting median (bold black horizontal line), minimum and maximum values (vertical “t” lines, or whiskers), and interquartile range (box). An asterisk indicates a significant difference according to Kruskal-Wallis one-way ANOVA (*P* < 0.05)

Table 2 shows the median, 25th percentile, and 75th percentile of iRAT, tRAT, and iAT. Quantitative analysis showed that the median score of tRAT was significantly higher than that of iRAT in each year. The median score of iAT was also significantly higher than that of iRAT in each year. The median score of tRAT was significantly lower than that of iAT in each year. The median iRAT, tRAT and iAT scores were significantly different across 2019–2021, as shown in Fig. 2.

**Table 2:** Comparison of LTBL scores for iRAT, tRAT, and iAT scores

	Total Attended	iRAT	tRAT	iAT	P ^a^	P ^b^	P ^c^
2015	60	75(72,77)	85(83,88)	95(93,97)	*P* < 0.001	*P* < 0.001	*P* < 0.001
2016	63	74(69,77)	81(79,83)	89(79,92)	*P* < 0.001	*P* < 0.001	*P* < 0.001
2017	62	74(71,79)	83(79,86)	92(88,95)	*P* < 0.001	*P* < 0.001	*P* < 0.001
2018	64	75(72,78.75)	81.5(79,85)	91.5(87,95.75)	*P* < 0.001	*P* < 0.001	*P* < 0.001
2019	61	73(71,74)	86(82,89)	94(89,95)	*P* < 0.001	*P* < 0.001	*P* < 0.001
2020	60	71(70,72)	81(80,81)	92.5(89,95)	*P* < 0.001	*P* < 0.001	*P* < 0.001
2021	63	74(71,77)	86(82,89)	94(89,97)	*P* < 0.001	*P* < 0.001	*P* < 0.001

Students’ scores reported as M (P25, P75)

^a^Indicates iRAT vs. tRAT

^b^Indicates iRAT vs. iAT

^c^Indicates tRAT vs. iAT

### Self-assessment

**Fig. 3:**
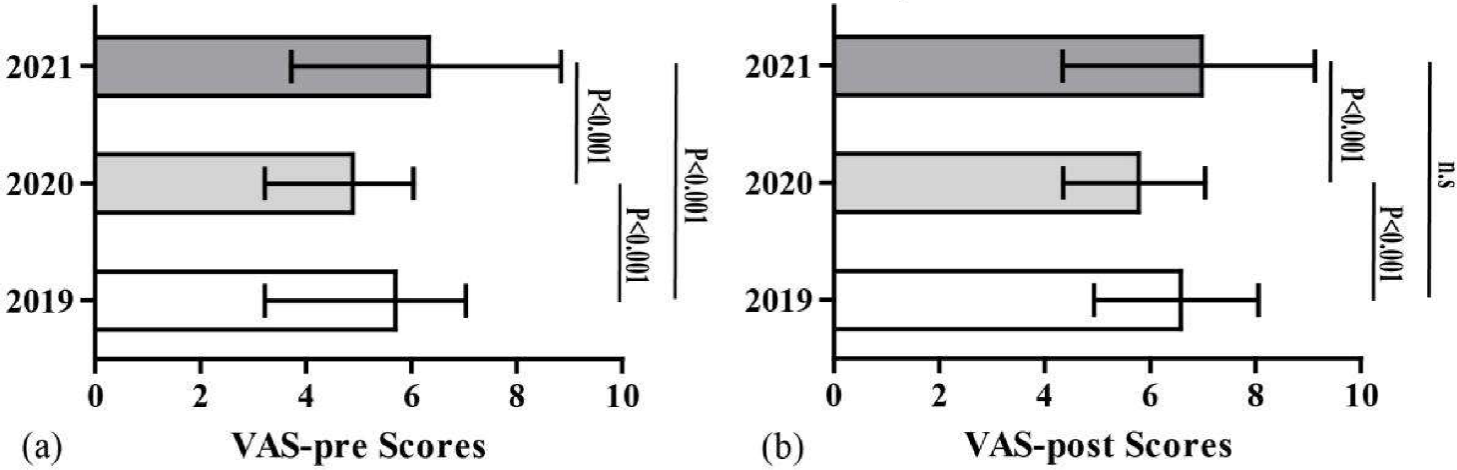
Histogram of the VAS-pre (**a**) and VAS-post (**b**) scores across Years 2019–2021, presenting minimum and maximum values (vertical “t” lines, or whiskers). An asterisk indicates a significant difference according to Kruskal-Wallis one-way ANOVA (*P* < 0.05)

Table 3 shows the mean and standard deviation of the VAS score (VAS-pre, the VAS test was carried out before TBL; VAS-post, the VAS test was carried out after the whole course). Statistical analysis showed that the mean score of VAS-post was significantly higher than that of VAS-pre in each year. The mean VAS-pre amd VAS-post scores were significantly different across 2019–2021, as shown in Fig. 3.

**Table 3:** Comparison of the Visual Analog Scale (VAS) score for the extent of students’ mastery of knowledge

	Total Attended	VAS-pre	VAS-post	P
2015	60	6.16 (0.92)	6.46 (0.12)	*P* < 0.001
2016	63	5.83 (1.08)	6.60 (1.16)	*P* < 0.001
2017	62	6.09 (0.88)	6.67(0.72)	*P* < 0.001
2018	64	6.11 (1.35)	6.39 (1.28)	*P* < 0.001
2019	61	5.69 (1.02)	6.57 (0.94)	*P* < 0.001
2020	60	4.89 (0.72)	5.78(0.74)	*P* < 0.001
2021	63	6.33 (1.12)	6.98 (1.20)	*P* < 0.001

### Questionnaire

The internal consistency and the validity of the questionnaire were confirmed. The Cronbach alpha coefficient for the questionnaire response was 0.963, indicating the questionnaire has a good internal consistency. Exploratory factor analysis showed an adequate validity (KMO = 0.873, *P* < 0.001). For quantitative analysis of the responses to the questions in the questionnaire, the following scores were used: strongly agree 5-4.20, agree 4.19–3.40, neutral 3.39–2.60, disagree 2.59–1.8, and strongly disagree 1.79-1.

Table 4 shows the mean scores for each statement regarding the first parameter (knowledge acquisition). The statement that had the highest score with 2015 and 2019 students was “LTBL makes students more motivated to study,” while the statement that had the highest score with 2016 and 2021 students was “It helps to get across the key points and difficulties in the design of removable partial dentures.” The statement that had the highest score with 2017 and 2018 students was “It enhances students’ ability to analyze and solve practical clinical problems with theoretical knowledge.” The statement that scored the highest with 2020 students was “It helps to memorize things for a longer time.”

**Table 4:** Mean and standard deviation of the first parameter (knowledge acquisition), according to the Likert scoring method, where SA represents strongly agree, A represents agree, and N represents neutral

Statements/mean (SD)	2015	2016	2017	2018	2019	2020	2021
LTBL increases students’ motivation to study.	4.32 (0.72) SA	4.31 (0.67) SA	4.29 (0.73) SA	4.14 (0.71) A	4.36 (0.68) SA	3.95 (0.79) A	4.22 (0.81) SA
It helps to get across the key points and difficulties in the design of removable partial dentures.	4.17 (0.69) A	4.33 (0.70) SA	4.22 (0.80) SA	4.28 (0.74) SA	4.24 (0.67) SA	4.05 (0.79) A	4.30 (0.75) SA
It enhances students’ ability to analyze and solve practical clinical problems with theoretical knowledge.	4.15 (0.78) A	4.13 (0.75) A	4.45 (0.67) SA	4.43 (0.69) SA	4.11 (0.78) A	4.17 (0.72) A	4.16 (0.87) A
It helps to memorize things for a longer time.	4.10 (0.82) A	4.17 (0.77) A	4.29 (0.66) SA	4.42 (0.69) SA	4.03 (0.77) A	4.27 (0.71) SA	4.27 (0.75) SA

Table 5 shows the mean scores for each statement in the second parameter (teamwork). The statement “8. It helps to develop critical thinking” had the highest score for 2015, 2016, and 2019, although the statement “It encourages students to work in teams to solve problems in the future” had the highest score for 2017 and 2020. In 2018, the highest score was obtained for the statement “LTBL helps to strengthen teamwork spirit and ability.” The statement “It encourages students to learn from each other” had the highest score for 2021.

**Table 5:** Mean and standard deviation of the second parameter (teamwork), according to the Likert scoring method, where SA represents strongly agree, A represents agree, and N represents neutral

Statements/mean (SD)	2015	2016	2017	2018	2019	2020	2021
LTBL helps to strengthen teamwork spirit and ability	3.67 (1.09) A	4.38 (0.68) SA	3.89 (1.11) A	4.53 (0.64) SA	3.67 (1.11) A	3.62 (0.83) A	3.83 (1.04) A
It enhances personal flexibility and respect for others	3.75 (0.91) A	3.63 (0.94) A	3.90 (1.17) A	3.91 (0.99) A	3.64 (0.95) A	3.55 (1.08) A	3.81 (1.09) A
It encourages students to learn from each other	4.32 (0.77) SA	4.37 (0.75) SA	4.00 (0.96) A	3.83 (0.98) A	4.25 (0.77) SA	4.00 (0.99) A	4.33 (0.70) SA
It helps to develop critical thinking	4.38 (0.76) SA	4.63 (0.63) SA	4.32 (0.70) SA	4.33 (0.89) SA	4.30 (0.76) SA	4.33 (0.75) SA	4.28 (0.63) SA
It encourages students to work in teams to solve problems in the future	3.90 (0.99) A	4.00 (0.97) A	4.45 (0.69) SA	4.27 (0.91) SA	3.93 (1.01) A	4.42 (0.67) SA	4.14 (0.76) A

Table 6 shows the mean scores for each statement in the first parameter (knowledge acquisition). The statement that had the highest score for 2015, 2017, 2018, and 2019 was “LTBL creates a more pleasant atmosphere in class,” while the statement that scored highest for 2016, 2020, and 2021 students was “It helps to pay more attention in class.”

**Table 6:** Mean and standard deviation of the third parameter (classroom atmosphere), according to the Likert scoring method, where SA represents strongly agree, A represents agree, and N represents neutral

Statements/mean (SD)	2015	2016	2017	2018	2019	2020	2021
LTBL creates a more pleasant atmosphere in class	4.25 (0.68) SA	3.67 (1.08) A	4.47 (0.72) SA	4.50 (0.69) SA	4.33 (0.68) SA	4.20 (0.82) SA	4.14 (0.78) A
It helps to pay more attention in class	4.08 (1.01) A	4.17 (0.89) A	4.34 (0.92) SA	4.47 (0.76) SA	3.98 (1.04) A	4.32 (0.79) SA	4.15 (0.87) A
It encourages students to attend classes and be more punctual	4.18 (0.87) A	4.10 (1.00) A	4.40 (0.83) SA	4.25 (0.85) SA	4.08 (0.88) A	4.20 (0.88) SA	4.03 (0.95) A
It improves students’ interactions	3.80 (1.13) A	3.87 (1.07) A	3.90 (0.88) A	3.86 (1.02) A	3.70 (1.13) A	3.80 (1.09) A	3.77 (0.97) A
It helps to communicate with teachers more conveniently and naturally	3.77 (1.20) A	3.83 (1.14) A	3.74 (1.08) A	3.86 (1.05) A	3.63 (1.20) A	3.87 (1.03) A	3.87 (0.81) A

Table 7 shows the mean scores for the additional statements regarding online LTBL (2020). All of the attending students (2020) strongly agreed with all of the additional statements. Overall, 43.3% of the attending students (2020) who watched the videos of online LTBL after class or when preparing for exams strongly agreed that “The videos help to review the content that I failed to catch up with at the online class and strengthen theoretical knowledge.” Table 8 shows the mean scores for the additional statements regarding the combination of online and in-class LTBL (2021). Overall, 30.2% of the attending students (2021) who watched the videos of online LTBL (2020) strongly agreed that “The videos help to review the content that I failed to catch up with at the online class and strengthen theoretical knowledge.”

**Table 7:** Mean and standard deviation of the additional statements for online LTBL (2020), according to the Likert scoring method, where SA represents strongly agree, A represents agree, and N represents neutral. Students who answered yes to “I reviewed the videos of online LTBL after class or when I was preparing for exams” would answer the last question

Statements/mean (SD)	2020
LTBL increases students’ attention in online classes	4.67 (0.57) SA
The online platform of LTBL is convenient	4.55 (0.53) SA
In the online LTBL classes, students can turn on the microphone at any time to ask the teacher questions, which enhances the interaction with the teacher	4.20 (0.80) SA
I reviewed the videos of online LTBL after class or when I was preparing for exams	Yes (43.3%), No (56.7%)
The videos help to review the content that I failed to catch up with in the online classes and strengthen theoretical knowledge	4.69 (0.47) SA

**Table 8:** Mean and standard deviation of the additional statements for the combination of online and in-class LTBL (2021), according to the Likert scoring method, where SA represents strongly agree, A represents agree, and N represents neutral. Students who answered yes to “20. I reviewed the videos of online LTBL (2020) after class or when I was preparing for exams” would answer the last question

Statements/mean (SD)/level	2021
I reviewed the videos of online LTBL (2020) after class or when I was preparing for exams	Yes (30.2%), No (69.8%)
The videos help to review the content that I failed to catch up with in the online classes and strengthen theoretical knowledge	4.26 (0.87) SA

## Discussion

This study evaluated students’ learning efficacy and their attitude towards LTBL via examination, self-assessment, and a questionnaire on topics regarding the design of removable partial dentures. LTBL includes three phases: Lecture-Based Learning, Team-Based Learning, and Clinical problem solving. This study also compared different methods of teaching LTBL, including in-class LTBL, online LTBL and both in combination. The two null hypotheses were rejected because the results indicated that students exhibited better learning performance after the LTBL course and students were satisfied with the LTBL course.

Repeated testing through iRAT, tRAT, and iAT during the LTBL course indicated the retrieval of new knowledge and the facilitation of knowledge recall [10]. As the course progressed, the scores of the three exams gradually improved in each academic year. In this study, students’ tRAT scores were significantly higher than their iRAT scores in each academic year, which suggested that the students showed more effective learning after the teachers’ lectures and discussions with their classmates. Similar results were confirmed in previous studies focused on dentistry students [1, 7, 9, 19]. Significantly higher scores were observed in the iAT examination than in the tRAT examination in each academic year, indicating that the knowledge acquired within the first two phases was consolidated and the ability to analyze and solve practical clinical problems was enhanced during the phase of clinical problem solving. Compared with iRAT scores, iAT scores were significantly enhanced, which illustrated that the whole LTBL course was beneficial to students’ learning of RPD design. VAS was used for students’ self-assessment of their prosthodontics knowledge. VAS-pre scores were significantly lower than VAS-post scores in each academic year, which indicated that the LTBL class enhanced the students’ mastery of prosthodontics knowledge.

Students in Year 2019 took in-class LTBL, students in Year 2020 took online LTBL due to the COVID-19 pandemic, and students in Year 2021 took the combination of in-class and online LTBL. iRAT, tRAT, VAS-pre, and VAS-post scores of students in Year 2020 were significantly lower than those in Years 2019 and 2021. This may be because the LTBL classes were taken online, which was associated with a class atmosphere and communication that were not as effective as in the in-class LTBL. This would be consistent with the findings of other studies [16, 20, 21]. iAT scores of students in Year 2021 were significantly higher than those in the other years, which may have been contributed to by the online video records.

Anonymous questionnaires were delivered to the students to evaluate their satisfaction with LTBL. The questionnaire consisted of 14 questions covering three parameters: knowledge acquisition, teamwork, and classroom atmosphere. In the first parameter (knowledge acquisition), numerous students agreed that the LTBL course could not only boost their study motivation but also help them obtain obscure knowledge and enhance their ability to analyze and solve practical clinical problems. These results were consistent with those for TBL courses organized in other studies [8, 22–24]. According to these studies, students preferred TBL to LBL because it created a more pleasant atmosphere and helped students to pay more attention in class, which was also confirmed in the third parameter (classroom atmosphere) of this study. Students agreed that LTBL helped them to be more punctual and improved their interaction with each other. Students could discuss and solve problems with their classmates rather than just listening to the teacher’s lecture. The LTBL course was intended to create a student-centered classroom, which could motivate students to solve clinical problems by themselves. However, some studies indicated that students become anxious about TBL courses, resulting from the need to prepare presentations and be evaluated by their peers [13–15]. In addition, students in this study agreed that the LTBL course made the communication between students and teachers more convenient and natural, expanding the students’ comfort and understanding zones. All of these high levels of satisfaction with the LTBL course should be attributed to the efforts of both teachers and students.

Regarding the second parameter (teamwork), students could realize their accountability for their own learning and identify the value of learning via discussion. Therefore, LTBL could encourage students to learn from each other and develop their critical thinking, with which most of the students agreed. Moreover, the study also indicated that LTBL helps to strengthen teamwork spirit and ability. Students worked hard to get high scores in tRAT, which encouraged friendly competition with other teams, in accordance with the findings in other TBL studies [25, 26]. The majority of students in this study were willing to solve problems through teamwork and their personal flexibility was also enhanced.

Students in Year 2020 had to study online due to the COVID-19 pandemic; however, the atmosphere and the efficacy of online classes are inferior to those of classes attended in-person. In this study, most of the students were satisfied with the online LTBL course and agreed that LTBL could increase their motivation to study. Moreover, their learning performance was significantly improved. Similar results were found in previous studies of online TBL [16, 20, 21, 27]. Students could proficiently use different online platforms and it was convenient for students to turn on the microphone at any time to ask the teacher questions, which enhanced the interaction with the teacher. However, some challenges still remain, including the reliability of internet networks and the organization and coordination ability of teachers. Overall, 43.3% of the students in Year 2020 and 30.2% of the students in Year 2021 reviewed the videos of online LTBL, which could help students to review the content that they failed to catch up with in the online class and strengthen their theoretical knowledge.

There are many branch disciplines in stomatology, involving orthodontics, periodontics, oral mucosal diseases, oral and maxillofacial surgery and other disciplines. These disciplines have similar characters: students are required to master complex theoretical knowledge; students have not yet engaged in clinical practice, which is difficult for them to form a systematic diagnosis and treatment thinking on the basis of theoretical knowledge [1]. Since the branch disciplines share common characteristics, their educational thinking is interlinked. In our study, LTBL exhibited excellent teaching efficacy in prosthodontics, which is one of the branch disciplines of stomatology. It can be speculated that the LTBL methodology also has potential to be applied in other branches of stomatology.

Since few studies have blended LBL with TBL in stomatology, we compared our study with other researches using TBL or LBL, respectively. Several studies have demonstrated that TBL was a highly structured collaborative format which augments student learning compared with LBL. It is reported that traditional lectures in stomatology can help students build solid knowledge but may make them boring due to few engagement [7]. Students in LBL class only receive knowledge instead of output knowledge, which is unfavorable for developing their critical thinking and doctor-patient communication skills [1]. Implementation of TBL in prosthodontics [1, 7–9, 28–30] and orthodontics [19] proved to enhance students’ clinical reasoning skills and motor skills, which reflected in greater student engagement with less demand on teachers’ contribution. However, TBL solely used in stomatology without LBL also has its deficiency, comprising weakness of the theoretical knowledge and mental burden of learning [12]. Thus, the LTBL methodology combines the advantages of both, and makes up for the shortage of LBL and TBL alone. It not only helps students understand and consolidate theoretical knowledge, but also trains students’ clinical diagnosis and treatment thinking.

To achieve the teaching objectives mentioned above, teachers are required to have rich clinical and teaching experience, and plan the course rationally. Before the formal class, teachers have to explain the whole course in detail. It is pivotal for students to understand the basic principles of the teaching methodology, clarify their responsibilities, and know what to expect so that they could actively engage in all phases of the LTBL course. In the TBL session, teachers should give specific instruction to group students and encourage their collaboration. It also takes teachers considerable effort to structure the iRAT, tRAT, and iAT exams, to make sure they covered the key knowledge and to direct the TBL in an efficient way. Therefore, extremely high request are are set to the instructor of LTBL. However, few teachers meet the requirements, which may limit the application of LTBL. In order to solve this problem, we can select high-level teachers and train teachers in various disciplines to make them clarify the details of LTBL methodology.

Between-course clinical practice takes place after the theoretical course and before the clinical rotation. It is the course of training students in simple clinical operation, which can be regarded as an important basis for students before entering clinical practice. Further studies may apply LTBL to between-course clinical practice and evaluate its applicability.

## Conclusion

Students at China Medical University were highly satisfied with the LTBL methodology in prosthodontics and their learning performance was improved as the course progressed, either online or in-class. Online LTBL could be delivered when students had to study online due to the COVID-19 pandemic; meanwhile, in-class LTBL performed better when combined with video records of the online LTBL course. This study supports the future application of LTBL in stomatology curricula, especially in demanding courses.

## Data Availability

Data is not publicly available but anonymized sets of data and replication files are available from the corresponding authors on request.

## Abbreviations

LTBL: Lecture-Team-Based Learning
LBL: Lecture-Based Learning
TBL: Team-Based Learning
COVID-19: Coronavirus Disease 2019
RPD: Removable partial dentures
iRAT: individual readiness assessment test
MCQs: multiple-choice questions
tRAT: team readiness assurance test
iAT: individual application test
VAS: Visual Analog Scales
